# Signal Existence Verification (SEV) for GPS Low Received Power Signal Detection Using the Time-Frequency Approach

**DOI:** 10.3390/s100504717

**Published:** 2010-05-10

**Authors:** Shau-Shiun Jan, Chih-Cheng Sun

**Affiliations:** Department of Aeronautics and Astronautics, National Cheng Kung University, Tainan 70101, Taiwan; E-Mail: p4896108@ccmail.ncku.edu.tw

**Keywords:** GPS signal acquisition, time-frequency analysis, low received power signal processing

## Abstract

The detection of low received power of global positioning system (GPS) signals in the signal acquisition process is an important issue for GPS applications. Improving the miss-detection problem of low received power signal is crucial, especially for urban or indoor environments. This paper proposes a signal existence verification (SEV) process to detect and subsequently verify low received power GPS signals. The SEV process is based on the time-frequency representation of GPS signal, and it can capture the characteristic of GPS signal in the time-frequency plane to enhance the GPS signal acquisition performance. Several simulations and experiments are conducted to show the effectiveness of the proposed method for low received power signal detection. The contribution of this work is that the SEV process is an additional scheme to assist the GPS signal acquisition process in low received power signal detection, without changing the original signal acquisition or tracking algorithms.

## 1. Introduction

Today, navigation is done worldwide, with the aid of the global positioning system (GPS) receivers. More than 400 million people worldwide rely on the satellite navigation to deliver the position, velocity, and time (PVT) information. At least four GPS satellites are required to provide a user position solution, which includes a position in three-dimensional coordinate and time information. The strength of received GPS signal transmitted from a satellite to the ground users is about −157 dBm below the noise floor of −138.5 dBm [1–3]. GPS is a direct-sequence spread-spectrum (DSSS) code-division-multiple-access (CDMA) system, and it can deal with the wideband noise and narrowband interference with low power contributed by the processing gain of the spreading code [4]. GPS receivers acquire and track the satellite signal using the correlation properties between the received satellite signal and the locally generated carrier and spreading code replicas. A maximum correlation value occurs, when the locally generated replicas align those of the received signal. However, the maximum correlation would be degraded due to the low received power GPS signal or the high power narrowband interference, especially in urban or indoor environments. It is because the received signal power might be blocked or shadowed by buildings or trees around the users. Accordingly, the number of acquired GPS satellites decreases extremely, such that the navigation performance is degraded or interrupted eventually. Researches focusing on the low received power GPS signal processing have attracted a lot of attentions in recent years [5–9].

Lin and Tsui in 2000 [5] compared several acquisition methods on criteria of receiver operation characteristics (ROCs), and suggested an approach, the non-coherent acquisition method, for weak signal acquisition. Psiaki in 2001 [6] reported a block acquisition method for weak GPS signal. The block acquisition method uses a predetection and integration time of 20 milliseconds, and subsequently formed 20 different accumulation parts, where each corresponds to a possible bit transition. As a result, this approach could acquire a weak signal with carrier-to-noise ratio (C/N_0_) as low as 21 dB-Hz. Yu and Zheng *et al.* in 2006 [7] proposed a differential combining (DFC) method for acquiring the weak GPS signal. The performance of the DFC method is superior to the conventional non-coherent approach [5] on the criteria of ROCs. The DFC could reduce the 3 dB processing loss of the non-coherent approach and improved the sensitivity by about 1.6 dB. The authors in 2009 [8] reported a weighted coherent overlapping (WCO) tracking method for low received power tracking. It could enhance the correlation value by coherent integration time method without reducing the Doppler window. In [9], the authors implemented the empirical mode decomposition (EMD) method to reduce the noise effect of the collected raw GPS signal. The signal-to-noise ratio (SNR) was therefore improved in the GPS signal acquisition process.

Most of the methodologies make efforts to enhance the acquisition performance based on the criteria of ROCs, which is a statistical process. For a satellite signal with low received power in the received GPS signal, the maximum correlation peak might be below the predetermined detection threshold, and it subsequently would be declared as signal absent. This paper therefore reports a solution called the signal existence verification (SEV) process to detect and verify whether the signal is absent. The SEV process is developed based on the time-frequency analysis [10], and it can detect the characteristic of the low received satellite signal on the generated spectrogram of the satellite signal. Furthermore, the SEV process could provide an additional stage for signal detection without changing the signal acquisition or tracking algorithms. The acquisition performance of the low received power GPS signal could be enhanced with the aid of the proposed SEV process, and the number of acquired GPS satellites could therefore be increased to continue the navigation service for users in urban or indoor environments. In addition, most of the applications of the time-frequency analysis in GPS field focused on the interference/jamming detection [11–13] or structural monitoring [14,15], and this work further extends the application of the time-frequency analysis on GPS signal detection.

Accordingly, the remainder of this paper is organized as follows. Section 2 presents the concept of the time-frequency analysis, and it includes the procedure of generating a spectrogram with small error. Section 3 reports the time-frequency applications on GPS low received power signal detection and verification. The concept of the signal existence verification (SEV) for low received power signals is explained in detail. In Section 4, several experiments are conducted to validate the performance of the proposed method. Finally, Section 5 presents the summary and concluding remarks.

## 2. Theoretical Development of the Time-Frequency Analysis

For a stationary signal characterized by time-invariant statistic properties, it can be adequately analyzed using well-known spectral techniques based on the Fourier transform (FT) [9]. For example, the Fourier filters can be employed to separate the noise from the real signal when the processes are linear and the noises have distinct time or frequency scales, which are different from those of the true signal [16]. However, the Fourier methods might fall short when the processes are either nonlinear or non-stationary. In fact, the GPS satellite signal with low received power might be “transient” in the receiver, and it therefore can be defined as a non-stationary signal since a non-stationary signal is “transient” in nature with duration generally shorter than the observation interval [9,16].

A concept of time-frequency representation had been proposed since 1950s [7], and it could be treated as an extension to Fourier analysis that is applicable to characterize signals whose spectral characteristics change with time. Current time-frequency representation tools, such as the Fourier spectrum, the short time Fourier transform (STFT or Gabor), the wavelet (Morlet) transform, or many high order schemes are generally implemented to the time-frequency analyses [7,17,18], require lower computational power and might be suitable for real-time applications, however the spectrogram generated might have poor time-frequency localization properties [11]. Another time-frequency analysis was represented by the Wigner–Ville distribution, which better localizes the signal on the time-frequency plane, but has the possible presence of cross-terms [11]. In general, a fast Fourier transform (FFT) algorithm could represent the spectrum of a signal in frequency domain. However, the resulting spectrum might have an exponentially decayed envelope if the signal is with a non-sinusoidal part [19]. As a result, several time-frequency tools, which are based on FT, cannot provide a precise spectrogram. This paper therefore implements a time-frequency analysis based on the Fourier sine spectrum to represent the information on a spectrogram with small error [19]. It could present almost all the frequency components of the received GPS signal on the corresponding spectrogram.

**Figure 1.**
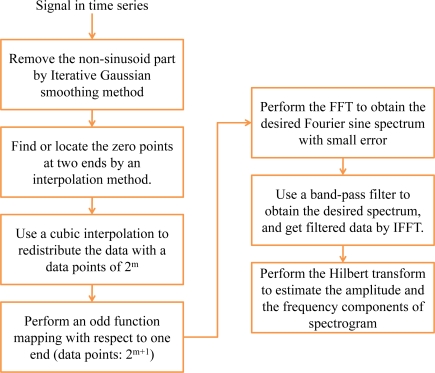
The procedure of generating a spectrogram with small error using the Fourier sine spectrum [19].

The procedure of generating a spectrogram with small error by the Fourier sine spectrum is depicted in Figure 1 [19]. Assume that a discrete signal can be approximated by [20]:

(1) $$y_{j}=\sum_{l=0}^{\infty }\left[b_{l}\;\text{cos}\;\frac{{2\pi t}_{j}}{\lambda_{l}}+c_{l}\;\text{sin}\;\frac{{2\pi t}_{j}}{\lambda_{l}}\right]+\sum_{n=0}^{N}a_{n}t_{j}^{n},\;\;\;\;\;-\infty <j<\infty$$

where the second summation represents the non-sinusoidal part and *N* is referred to as the largest power for which *a_n_* → 0 for all *n* ≥ *N*. Note that the non-sinusoidal part is interpreted as the sum of monotonic part and all the Fourier modes whose wavelength is longer than the expansion interval. The collected signal is input to an iterative Gaussian smoothing filter, which can remove the non-sinusoidal components for grasping pure spectral information of the input signal [20]. After the iterative Gaussian smoothing method by *m* times, where *m* ≥ *N*/2, the corresponding results can be rearranged in the following [19,20]:

(2) $$y_{m}^{\prime }=\sum_{l=0}^{\infty }{\left[1-\text{exp}\left(\frac{-2\pi^{2}\sigma^{2}}{\lambda_{l}^{2}}\right)\right]}^{m}\left[b_{l}\;\text{cos}\;\frac{2\pi t}{\lambda_{l}}+c_{l}\;\text{sin}\;\frac{2\pi t}{\lambda_{l}}\right]$$

(3) $$\bar{y}(m)=y-y_{m}^{\prime }=\sum_{l=0}^{N}A_{l,m,\sigma }\left[b_{l}\;\text{cos}\;\frac{2\pi t}{\lambda_{l}}+c_{l}\;\text{sin}\;\frac{2\pi t}{\lambda_{l}}\right]$$

where $y_{m}^{\prime }$ and *ȳ_m_* are the high frequency and smoothed parts at the *m*^th^ smoothing step, respectively, and *σ* is the smoothing factor. As a result, the non-sinusoidal part is removed completely. Note that, for a finite data, the result of applying the Gaussian smoothing method is just the zero-order least squares response of the data. For the interior points, the resulting smoothed response is approximately diffusive with a wide transition zone around the cutoff frequency selected to filter a given dataset. The iterative Gaussian smoothing method is therefore used to repeatedly smooth the remaining high frequency part. It is effective to remove a non-sinusoidal polynomial with finite degree [20]. Moreover, the whole procedure can be performed in the spectral domain whose operation count is slightly more than twice of the use of the FFT. Subsequently, the zero points around the two ends are identified by a searching procedure and an interpolation method. The desired data, which is between two zero points, is redistributed by a cubic interpolation method to a data with a length of 2^m^. Performing an odd function mapping of the redistributed data, the data length is of 2^m+1^.

Consequently, the corresponding Fourier sine spectrum could be therefore obtained by performing the FFT. The resulting Fourier sine spectrum is a slightly better approximate parameter representation of the sinusoidal part than that of the original Fourier spectrum. Specifically, the Fourier sine spectrum is a projection in strong sense while the Fourier spectrum is a projection in weak sense, such that the resolution of minor modes of the former is better than that of the latter [20]. Choosing a band-pass filter to filter out the signal at the desired frequency, the signal in time domain is subsequently obtained by performing the inverse FFT (IFFT). For a sinusoidal signal, *y*(*t*), defined in the range of −∞ ≤ *t* ≤ ∞, the following Hilbert transform is the corresponding imaginary part [21]:

(4) $$\tilde{y}(t)=\int_{-\infty }^{\infty }\frac{y(\tau )}{\pi (t-\tau )}d\tau$$

The amplitude and frequency can be evaluated directly as follows:

(5) $$\begin{array}{l} z(t)=y(t)+i\tilde{y}(t)=A(t)e^{i\theta (t)}\\ A(t)=\sqrt{y^{2}(t)+{\tilde{y}}^{2}(t)}\\ \theta (t)=\;{\text{tan}}^{-1}(\frac{\tilde{y}(t)}{y(t)})=2\pi f_{0}t \end{array}$$

**Figure 2.**
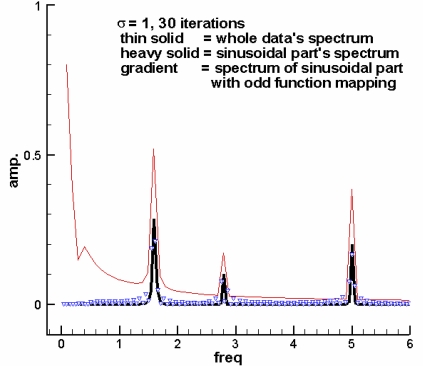
An example of the non-sinusoidal part effect in Fourier spectrum (adopted from [22]).

Finally, the corresponding imaginary part, and subsequently the amplitude and the frequency information, can be estimated based on the Hilbert transform [21]. As a result, the spectrogram with small error is obtained. From the resulting spectrogram much information concerning the dominant modes and corresponding harmonics can be captured. Moreover, the associated amplitude and frequency variations are partially reflected by the spectrum scattering around a mode. Thus, the detailed variations can be obtained by examining the spectrogram. In other words, it could provide an effective tool for detecting the low received power GPS signals by means of the two dimensional time-frequency analysis. Figure 2 shows an example of the non-sinusoidal part effect. The thin solid line represents the Fourier spectrum of the original signal with a non-sinusoidal part. The heavy solid line indicates the Fourier spectrum of the sinusoidal part. It shows that the spectrum of the original signal has the error in the low frequency part because of the non-sinusoid part signal. The gradient line depicts the resulting spectrum of the sinusoid part via iterative Gaussian smoothing method with odd function mapping. It is important to note that the odd function mapping in step 4 ensures that the periodicity of the signal is valid up to the highest order derivative of the data [19]. Consequently, the spectrum error can be effectively reduced.

## 3. The Signal Existence Verification (SEV) Process for GPS Signal Detection

**Figure 3.**
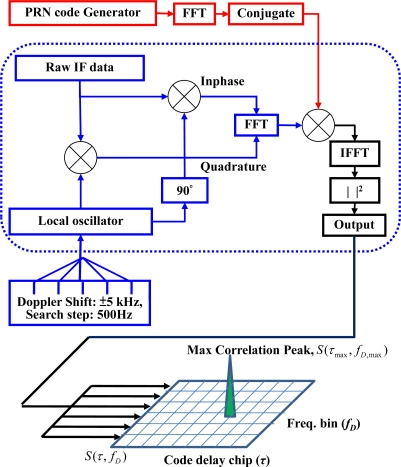
The GPS signal acquisition process using the parallel code phase search acquisition method.

This paper focuses on the GPS signal transmitted on L1 frequency band (1575.42 MHz). After down-conversion in the front-end and sampling by an A/D converter, the GPS L1 signal received from the *k*^th^ satellite, $S_{L1}^{\left(k\right)}[n]$, can be described as [23]:

(6) $$S_{L1}^{\left(k\right)}[n]=\sqrt{P_{c}}C^{\left(k\right)}[n]D^{\left(k\right)}[n]\text{cos}[\omega_{\mathrm{IF}}n]+e[n]$$

where *P_c_* is the power of signal associated with C/A code. *C*^(*k*)^ is the 1023-chip coarse/acquisition (C/A) code sequence with a chip rate of 1.023 MHz assigned to satellite number *k*. The C/A code is also called the pseudorandom noise (PRN) code, and each PRN number could represent an identification of a specific GPS satellite [1–3]. *D*^(*k*)^ is the navigation data sequence of a binary value with a data rate of 50 Hz assigned to satellite number *k*. *ω_IF_* is the intermediate frequency (IF) after front-end down-conversion, and *n* denotes the *n*^th^ sample of signal in discrete time representation. *e*[*n*] is the undesired signal, which might consist of interference or Gaussian noise. The signal detection process of a GPS receiver is implemented in the signal acquisition stage, which estimates the PRNs, code delay chips, and Doppler frequency of satellites in the received GPS signal. It is required to search the possible Doppler frequency shift and code delay chip in the range of ±5 kHz and 0–1023 chips, respectively [1–3,24]. In general, the conventional acquisition process implemented in a software defined receiver (SDR) utilizes the parallel code phase search acquisition method [24], as shown in Figure 3. The correlation process between the GPS raw IF signal and the locally generated replica is done based on a method of performing circular correlation through Fourier transform [3,25]. The incoming GPS signal is first multiplied by a locally generated carrier signal. Multiplications with the carrier signal and a 90° phase shifted version of the carrier signal generate the inphase (I) and quadrature (Q) signals, respectively. The I and Q signals are combined to form a complex input signal, *x*[*n*], to the FFT function, and *x*[*n*] can be expressed as

(7) x[n]=I[n]+jQ[n]

The generated PRN code is transformed into the frequency domain by FFT and the result is complex conjugated. The Fourier transform of the input GPS signal is subsequently multiplied with the Fourier transform of the PRN code. The multiplication result is transformed into the time domain by an IFFT. As a result, the absolute square value of the output of the IFFT represents the correlation result between the input GPS signal and the local replica of a specific PRN. The correlation result is a two-dimensional search pattern, *S*(*τ*, *f_D_*), called the ambiguity function, which is a function of the code delay chip *τ* and Doppler frequency bin *f_D_* [1,2].

**Figure 4.**
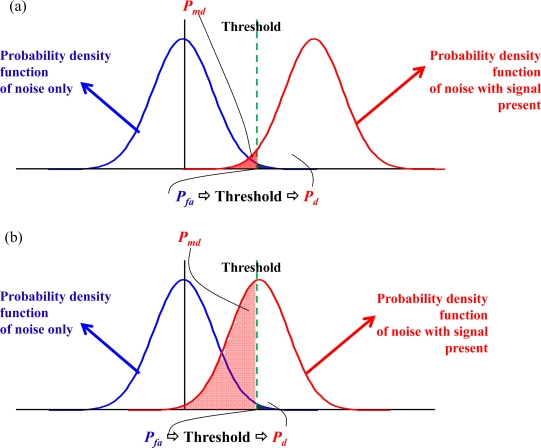
(a) The concept of typical strength GPS signal detection in acquisition process. (b) The concept of low received power GPS signal detection in acquisition process.

Each cell of the search pattern represents the correlation value at the corresponding code delay chip and Doppler frequency bin. If a correlation peak *S*(*τ*_max_, *f*_*D*,max_) can be found on the search pattern, the index (*τ*_max_, *f*_*D*,max_) of this peak denotes the code delay sample and the Doppler frequency for the specific PRN (*i.e.,* satellite). With the acquired information, the receiver tracks and subsequently decodes the navigation bits, and the user position can be estimated accordingly [1–3,23]. In general, a SNR of a satellite signal can be estimated from the maximum correlation peak [1–3,24]. In theory, the signal detection in the acquisition process is a statistical process, because each cell either contains noise with the signal absent or noise with the signal present [2]. Figure 4(a) shows an example of a single trial decision where the blue and red curves represent the probability density functions (pdfs) of noise with signal absent and present, respectively. The pdf for noise with signal absent has a zero mean, and the pdf for noise with signal present has a non-zero mean. A satellite signal is declared present if the estimated SNR in the acquisition process is beyond the threshold. However, a false alarm of signal present might occur as the blue area of Figure 4(a). The signal detection process would have a probability of miss-detection (*P_md_*) as shown in the red area of Figure 4(a). The false alarm probability (*P_fa_*) is defined under the hypothesis *H*_0_ of signal absence or code delay/Doppler frequency shift mismatch as [1,2]:

(8) $$P_{\mathrm{fa},V_{t}}=P_{\mathrm{fa}}\left(S(\tau_{\text{max}},f_{D,\text{max}})>V_{t}|H_{0}\right)$$

It is the probability that the decision variable *S*(*τ*_max_, *f*_*D*,max_) passes a fixed threshold *V_t_* under *H*_0_. On the contrary, the detection probability (*P_det_*) is defined under the hypothesis *H*_1_ of signal presence and perfect delay and frequency alignment between the received signal and the local replica [1,2]:

(9) $$P_{\text{det},V_{t}}=P_{\text{det}}\left(S(\tau_{\text{max}},f_{D,\text{max}})>V_{t}|H_{1}\right)$$

In general, a required false alarm probability (*P_fa_*) is chosen first, and the corresponding threshold and detection probability (*P_d_*) can be estimated [2].

**Figure 5.**
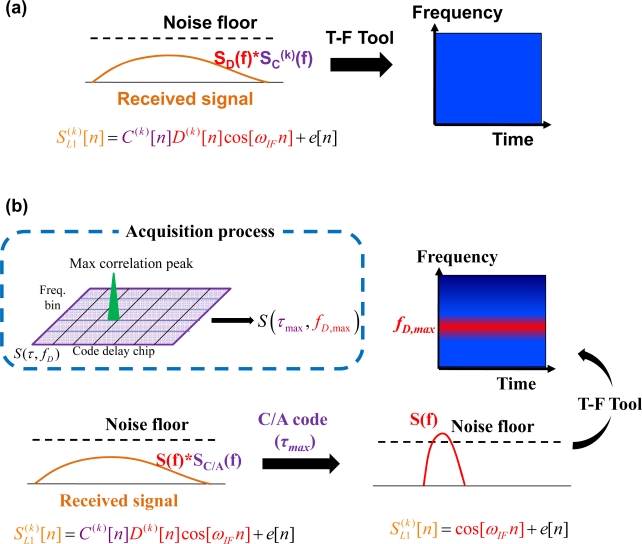
(a) An example of mapping a raw GPS signal into the time-frequency plane by a time-frequency tool. (b) The concept of satellite signal detection by using the time-frequency tool.

In addition, the GPS signal processing is sensitive to noise. For the low received power satellite signal case, the pdfs curves of noise with signal absent and present are close as shown in Figure 4(b). The probability of miss-detection would be increased while the required false alarm probability is strict as that of the signal with a typical strength. As a result, the satellite signal with low received power might be declared as signal absent while the acquired SNR is below the threshold. The number of acquired satellite therefore decreased and then might not be sufficient for positioning. This paper therefore designs a scheme, called the signal existence verification (SEV) process, based on the time-frequency approach to verify whether the satellite signal is absent. Note that the power of GPS signals is spread over a much wider bandwidth than that of the original information because GPS is a DSSS system [4]. As a result, the GPS signals present power spectral densities are completely hidden under the noise floor, as shown in the left plot of Figure 5(a). In other word, it is difficult to observe the useful information of satellite signals on the spectrogram if the GPS raw signal is mapped into the time-frequency plane directly, as shown in the right plot of Figure 5(a). Thanks to the processing gain of the spreading code, the power spectral density of original information could be despread beyond the noise floor. The proposed SEV method therefore takes the characteristic to perform the verification of the low received power satellite signal, as shown in Figure 5(b). The C/A code effect is removed from the received satellite signal by multiplying a local replica C/A code generated according to the acquired code delay information, *τ*_max_, in the acquisition process.

From Equation (1), if the signal is present, the remaining signal with a length of 1 millisecond would consist of a carrier and undesired noise terms as:

(10) $$S_{L1}^{\left(k\right)}[n]=\;\text{cos}[\omega_{\mathrm{IF}}n]+e[n]$$

**Figure 6.**
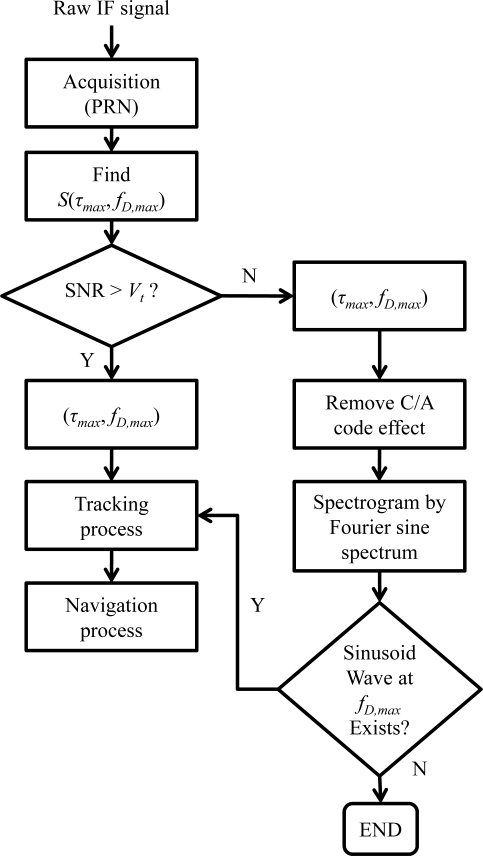
The block diagram of the signal existence verification (SEV) process for the low received power GPS signal detection.

Note that the navigation data is the same value within 1 millisecond. The remaining signal is subsequently mapped into the time-frequency plane by a time-frequency tool. Ideally, the characteristic of the sinusoidal wave can be further detected around the Doppler frequency of *f_D,max_* from the resulting spectrogram, as shown in the time-frequency plane of Figure 5(b). On the contrary, it would be difficult to observe the helpful information in the generated spectrogram if the satellite signal is absent. That is, the original satellite signal information could not be despread out with the wrong code delay information. In reality, the power of the despread sinusoid wave might be close to the noise floor and difficult to observed from the time-frequency plane on the condition of the low received power signal. As discussed in Section 2, the time-frequency method based on the Fourier sine spectrum could therefore provide an effective tool for the low received power detection. A detailed block diagram of the SEV process for a specific PRN (satellite) signal is shown in Figure 6. In addition, the SEV process would be executed after the acquisition process while the acquired SNR of a satellite signal is below and close to the threshold *V_t_*.

## 4. Experiment Results and Analyses

Several experiments were conducted to validate the proposed method. This paper used a software defined GPS receiver to collect real GPS signals including the typical received power satellite signals and the low received power satellite signals for evaluating the performance of the proposed SEV method. The typical received power (outdoor) GPS signal was collected on the roof of the Department of Aeronautics and Astronautics building of National Cheng Kung University, Taiwan. In addition, the low received power (indoor) GPS signal was collected in room 5896 of the same building. Both outdoor and indoor signals were collected at the same time on January 22th, 2010. In other words, the in-view satellites of both signals would be similar. The in-view satellites of the roof GPS signal could be regarded as a true solution for the low received power satellite signal detected by the proposed method. The sampling frequency and IF of the collected GPS data was 16.368 MHz and 4.1304 MHz, respectively. The collected IF samples are stored in sign of 8 bits per sample. In this paper, the SNR of the acquired GPS satellite signal is calculated according to the correlation peak to next peak ratio (CPPR) method [26]. The detection threshold of this work was chosen as 3.5 dB [27]. In the following analyses, each acquisition process takes the GPS signal with data length of 1 millisecond (*i.e.,* a complete sequence of C/A code in one period), and so does the SEV process. The frequency resolution of the spectrogram is 83.3 Hz because the Doppler search band of 10K Hz is divided into 120 bins (*i.e.,* 10,000 Hz/120 = 83.33 Hz).

**Figure 7.**
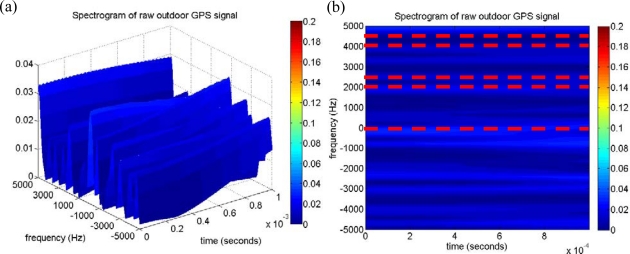
(a) The spectrogram of the raw outdoor GPS signal by using the time-frequency tool. (b) The top view of (a), several continuous waves could be observed in the time-frequency plane.

**Figure 8.**
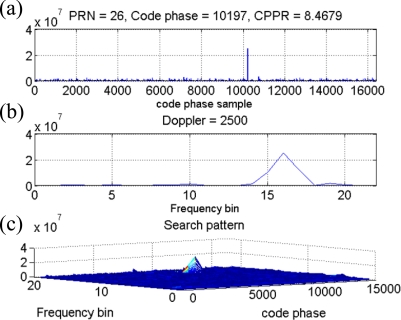
(a) The correlation distribution *versus* the code delay samples of PRN 26 at the frequency bin with maximum correlation peak. (b) The correlation distribution *versus* the frequency bin of PRN 26 at the code delay sample with maximum correlation peak. (c) The search pattern of the PRN 26 of the outdoor collected signal in the acquisition process.

**Figure 9.**
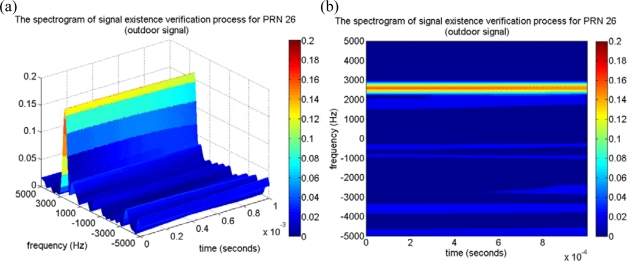
(a) The spectrogram of the signal existence verification (SEV) process for PRN 26 satellite signal. (b) The top view of (a), a characteristic sinusoid wave can be observed around the frequency of 2,500 Hz.

In the following analysis, the collected typical received power GPS signal is investigated by the time-frequency approach to validate the SEV process. The collected outdoor signal is acquired using the conventional acquisition method with the integration time of 1 millisecond, and the acquired satellites information is listed in Table 1 including the PRNs, the code delay samples, the Doppler frequency, and the estimated CPPRs. Note that several PRNs are located at the similar Doppler frequency. For example, the Doppler frequencies of PRNs 22 and 30 are similar, and so are those of PRNs 12 and 18. After applying the time-frequency method to the same outdoor GPS signal, Figure 7 represents the resulting spectrogram, and it also demonstrates that the GPS signal present power spectral densities are completely hidden under the noise floor as mentioned in Section 3. Figure 7(b) is the top view of Figure 7(a), and some weak continuous wave information can be observed from the resulting spectrogram but that might not be contributed by the GPS satellite signals. The in-view satellite signals are marked on the spectrogram at their corresponding Doppler frequency as the dash lines shown in Figure 7(b), and it is difficult to obtain the valid relationship between the information gained from the resulting spectrogram and the in-view satellite signals. Consequently, we would not be able to directly detect the GPS satellite signals from the spectrogram of the raw GPS signal. However, a characteristic of the satellite signal is observed when the SEV process is implemented. An example is represented by the same outdoor satellite signal of PRN 26. The corresponding acquired results such as the correlation distribution with the maximum code delay and Doppler bin are shown in Figures 8(a) and 8(b), and the search pattern is shown in Figure 8(c). The estimated CPPR of PRN 26 is 8.47 dB, which is well beyond the threshold of 3.5 dB, and an obvious maximum correlation peak is expected on the search pattern of Figure 8(c). In addition, Figure 9 illustrates an example of PRN 26 utilizing the SEV process to verify whether the satellite signal is definite present. According to the procedure of SEV process, the C/A code effect is removed by multiplying the locally generated C/A code with a code delay of 10,197 samples. The remaining signal is subsequently mapped into the time-frequency representation by the time-frequency tool described in Section 2. By the SEV process, a characteristic of the sinusoid wave should be observed locating around the frequency of 2,500 Hz while the satellite signal of PRN 26 is included in the received GPS signal. In the resulting spectrogram of Figure 9(b), a characteristic of the sinusoid wave is observed around the Doppler frequency of 2,500 Hz, which matches the acquired information of PRN 26 in Table 1. Note that drift rate of the Doppler frequency within 1 millisecond is not large so that the observed characteristic of the sinusoid wave is almost unchanged on the spectrogram. As a result, the SEV process is validated for the typical received power GPS satellite signal, and it provides an additional evidence to confirm the satellite signal of PRN 26 exists in the collected outdoor GPS signal.

The following experiments demonstrates the significant improvements that the SEV process brings to detection of the low received power GPS satellite signal, and the SEV process will be applied to the satellite signal with acquired SNR below the threshold of a general acquisition process. Firstly, Table 2 shows the acquisition results of the collected indoor signal by the conventional acquisition method with the integration time of 1 millisecond. Note that the PRNs listed in Table 2 are the satellites in view according to that of the collected roof GPS signal (in Table 1). The background color in gray of Table 2 indicates that the acquired CPPRs are below the threshold. As a result, only the satellite signal of PRN 30 is able to be acquired from the indoor signal. The conventional acquisition method with the coherent and non-coherent method with the integration time of 2 milliseconds is implemented to acquire more possible satellites in view, and the results are shown in Table 3. However, PRN 30 is still the only satellite with SNR beyond the threshold.

**Figure 10.**
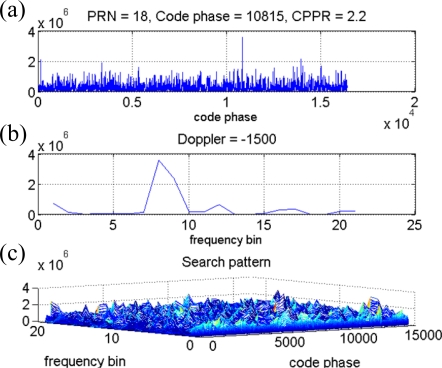
(a) The correlation distribution *versus* the code delay samples at the frequency bin with maximum correlation peak. (b) The correlation distribution *versus* the frequency bin at the code delay sample with maximum correlation peak. (c) The search pattern of the PRN 18 of the collected indoor signal in the acquisition process.

**Figure 11.**
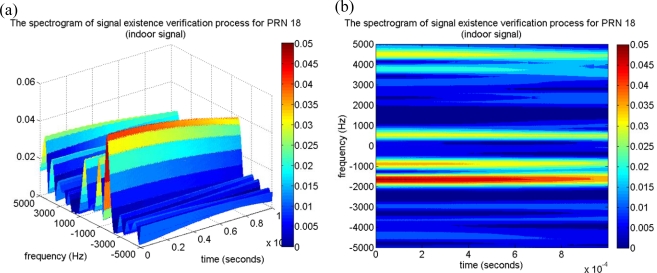
(a) The spectrogram of the proposed approach for PRN 18 satellite signal. (b) The top view of (a), a characteristic sinusoid wave can be observed around the frequency of −1,500 Hz.

It is as expected that the number of acquired satellites decreases extremely under the indoor environment, but the satellite signal which is declared as absent by the acquisition process might be present in reality. Accordingly, the proposed approach was applied to a satellite signal whose CPPR is below the threshold to verify whether it is absent. This work takes the satellite signal of PRN 18 as an example, because PRN 18 was located on the window-side of room 5896 when the experiment was conducted, and it is possible to be received by the indoor GPS receiver. As indicated in the conventional acquisition result, it is difficult to observe an obvious maximum correlation peak on the corresponding search pattern, as shown in Figure 10(c), and it is declared as signal absent because the acquired CPPR of PRN 18 is 2.20 dB, which is below the threshold of 3.5 dB. The correlation distribution with the maximum code delay and Doppler bin of the PRN 18 are shown in Figures 10(a) and 10(b), respectively. Through the conventional acquisition method, although PRNs 18 is declared as absent, the PRN 18 signal might be in the collected indoor GPS signal. The proposed approach is therefore implemented to verify the presence of the PRN 18 signal. The proposed approach firstly removes the C/A code effect by multiplying it with the locally generated C/A code with a code delay of 10,815 samples. Applying the time-frequency tool to map the remaining signal onto the time-frequency plane, the resulting spectrogram is shown in Figure 11. It is important to note a characteristic of sinusoid wave is observed around the Doppler frequency of −1,500 Hz, which matches the acquired information of PRN 18 in Table 2. Although the strength of the characteristic of the sinusoid wave is not as obvious as that of the typical strength satellite signal in Figure 9, the PRN 18 signal is detectable around the corresponding Doppler frequency on the spectrogram. The attenuation is expectable for a satellite signal with a low received power. That is, although the satellite information is despread with a spreading gain, it might be close to the noise floor. In other words, the proposed approach could provide evidence to show that the satellite signal of PRN 18 is present. In reality, the satellite signal of PRN 18 is in view according to the acquisition result of the collected roof GPS signal, as shown in Table 1. The SEV process further provided evidence that the PRN 18 is present, although it was previously categorized as noise by the acquisition process.

After applying the proposed SEV process to the others satellite signals, which were previously declared as signal absent in Table 3, five additional satellites (PRNs), which are marked in red in Table 4, are now declared as signal present. As a result, the SEV process increases the number of acquired satellites from one to more than four, that is, it meets the basic requirements (*i.e.*, at least four satellites in-view) for continuing the navigation service under indoor environments. Consequently, the proposed SEV process is effective for detecting the satellite signals with low received power.

**Figure 12.**
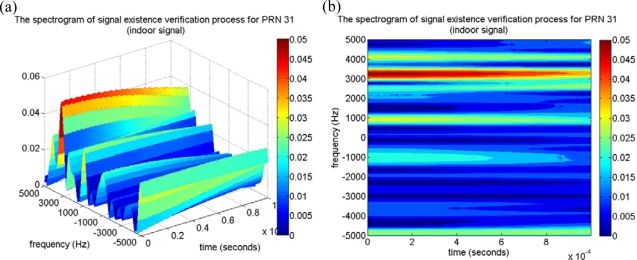
(a) The spectrogram of the proposed approach for PRN 31 satellite signal. (b) The top view of (a), a characteristic sinusoid wave can be observed around the frequency of 3,000 Hz.

**Figure 13.**
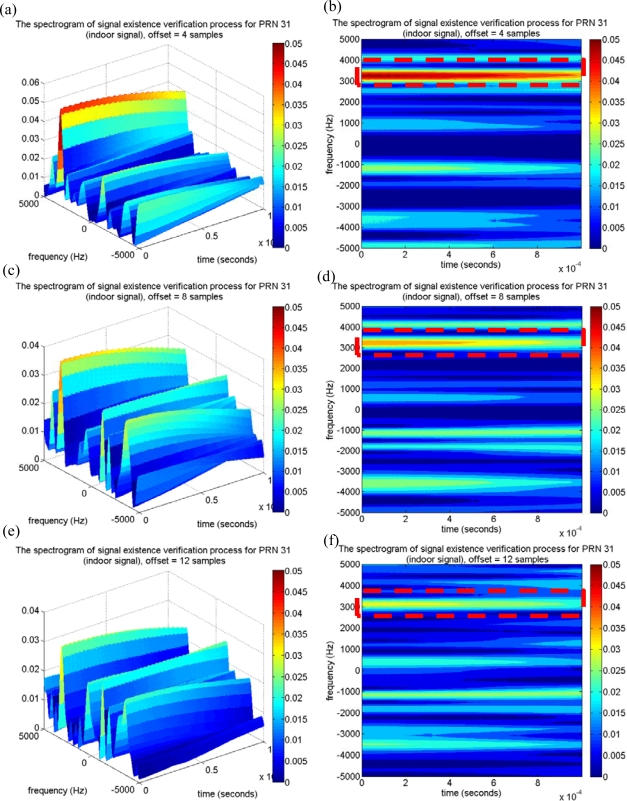
(a) The spectrogram of the proposed approach for PRN 31 satellite signal with miss-alignments of four samples. (b) The top view of (a). (c) The proposed approach for PRN 31 satellite signal with miss-alignments of eight samples. (d) The top view of (c). (e) The proposed approach for PRN 31 satellite signal with miss-alignments of 12 samples. (f) The top view of (e).

For the satellite signal with low received power, the code delay sample of the maximum correlation peak estimated in the acquisition process might not be exactly correct. The miss-alignment between the code delay of the local C/A replica and that of the incoming satellite signal would result in the imperfect removal of C/A code effect, and the maximum correlation value and its resulting SNR would be therefore degraded. This paper also investigates the performance of the proposed approach if the estimated code delay sample has an offset from the correct one. The satellite signal of PRN 31 is used in this work as an example, because PRN 31 was also located on the window-side of room 5896 when the indoor experiment was conducted, and the PRN 31 signal might be received by the indoor GPS receiver. Similarly, the proposed approach removes the C/A code effect by multiplying it with the locally generated C/A code with a code delay of 6,178 samples. The time-frequency tool is then applied to map the remaining signal onto the time-frequency plane, and the resulting spectrogram is shown in Figure 12. Note that a characteristic sinusoid wave is detected around the Doppler frequency of 3,000 Hz. The strength of the characteristic is stronger than that of PRN 18, and this result agrees with the acquired CPPRs in Table 2. Again, the proposed approach verifies that the satellite signal of PRN 31 is present, although the satellite was categorized as signal absent by the conventional acquisition process. Moreover, the same PRN 31 signal, but with the offsets of 4, 8, and 12 samples from the correct code delay of 6,178 samples, were also tested to show the effectiveness of the proposed approach for the low received power satellite signal detection. By the similar procedures of the proposed approach, the C/A code effect is partially reduced by multiplying it with the locally generated C/A code which is miss-aligned with the incoming signal by the above offsets, and the resulting spectrograms are shown in Figure 13. As shown in the results, the strength of the characteristic of the sinusoid wave decreases as the number of offset samples increases, as shown in the red dashed line box of each spectrogram in Figure 13. For the case of the low received power satellite signal, it is important to note that the strength of the characteristic of the sinusoid wave is sufficient for signal detection even if the miss-alignment between the local C/A code replica and that of the incoming satellite signal is half a chip (*i.e.,* eight samples). However, the characteristic of the sinusoid wave is undetectable on the spectrogram if the miss-alignment is larger than three-quarters of a chip (*i.e.*, 12 samples), as shown in Figure 12(f). Consequently, the proposed approach is capable of detecting and verifying the satellite signal with low received power even if the estimated code delay information has an offset of half a chip (eight samples) from the correct one.

In this paper, the computation load of the SEV process is also investigated. Note that the time-frequency representation of the SEV process dominates the computation load. Furthermore, the resolution of the time-frequency plane depends on the number of data samples in the time and frequency bins. For this reason, the computation load of the time-frequency representation according to the resolution in time series and frequency bins is studied. In this paper, the GPS receiver with a sampling frequency of 16.368 MHz is used to collect the raw GPS signal. The resolution of time-frequency plane in this paper was chosen as 83.33 Hz (*i.e.*, ±5 kHz divided into 120 bins) in frequency and 61.09 ns (1 ms divided by 16,368 samples) in time. Accordingly, the SEV process requires 122.4 seconds to complete the time-frequency representation. That is, each frequency bin with a number of 16,368 samples in time consumes 1.02 seconds, as shown in Table 5. For a practical issue, a trade-off could be made between the time-frequency resolution and the computation load. It is possible to implement a down-sampling process to reduce the samples of the data stream prior to the SEV process. In addition, the reduction in the Doppler window and the number of frequency bins according to the acquired Doppler information will be able to lower the computation load as well. Consequently, this paper also examines the computation loads for possible combinations of samples in time, as depicted in Table 5.

## 5. Conclusions

The strength of the received GPS signal is lower for urban or indoor environments because of multipath or sheltered effects from the neighboring buildings or trees. This might result in degraded navigation accuracy or complete loss of receiver tracking. This paper proposed a signal existence verification (SEV) process for low received power GPS signal detection based on the time-frequency analysis, which could generate a spectrogram with small error. As shown in the results of this paper, the SEV process can successfully verify the presence of one satellite signal even if the acquired SNR of that specific satellite is below the threshold. The proposed SEV process first removes the C/A code effect from the original signal according to the acquired code delay information in the acquisition process. The spectrogram of the remaining signal is subsequently generated to observe the characteristic of a sinusoid wave at the corresponding Doppler frequency. The experimental results also demonstrated the effectiveness of the proposed SEV process in the low received power GPS signals. Furthermore, the proposed approach is capable of detecting and verifying the satellite signal with low received power even if the estimated code delay information has an offset of half a chip from the correct one.

## Figures and Tables

**Table 1.** The acquisition result of the collected outdoor GPS signal by the conventional acquisition method with the integration time of 1 millisecond.

**PRN**	**Code delay samples**	**Doppler frequency (Hz)**	CPPR* (dB)
22	13,351	2,000	12.00
30	8,976	2,000	11.40
14	13,088	4,500	8.62
26	10,197	2,500	8.47
18	5,597	0	8.03
12	7,544	0	5.78
31	12,492	4,000	5.25

* CPPR: Correlation peak to next peak ratio

**Table 2.** The acquisition result of the collected indoor signal by the conventional acquisition method with the integration time of 1 millisecond.

**PRN**	**Code delay samples**	**Doppler frequency (Hz)**	CPPR* (dB)
22	1,419	1,000	0.85
30	2,543	2,500	7.55
14	2,954	1,500	1.14
26	15,588	−1,500	1.52
18	10,815	−1,500	2.20
12	7,445	500	1.17
31	6,178	3,000	2.61

* CPPR: Correlation peak to next peak ratio

**Table 3.** The acquisition result of the collected indoor signal by the conventional acquisition method with the coherent and non-coherent integration time of 2 milliseconds.

**PRN**	**Code delay samples**	**Doppler frequency (Hz)**	CPPR* (dB) by coherent	CPPR* (dB) by non-coherent
22	6,923	−1,500	1.24	1.17
30	2,541	2,500	8.05	8.04
14	16,183	2,500	1.97	2.01
26	16,172	1,000	2.68	2.67
18	1,845	2,000	0.35	0.32
12	15,207	1,000	0.55	0.50
31	14,774	2,500	0.53	0.49

* CPPR: Correlation peak to next peak ratio

**Table 4.** The acquisition result of the collected indoor signal by using the conventional acquisition method with 1 ms integration time and applying the SEV process.

**PRN**	**Code delay samples**	**Doppler frequency (Hz)**	CPPR* (dB)
22	1,419	1,000	0.85
30	2,543	2,500	7.55
14	2,954	1,500	1.14
26	15,588	−1,500	1.52
18	10,815	−1,500	2.20
12	7,445	500	1.17
31	6,178	3,000	2.61

* CPPR: Correlation peak to next peak ratio

**Table 5.** The computation cost of the SEV process.

**Sampling frequency (MHz)**	**Total samples**	**Consumption time/bin (Second)**
16.368	16,368	1.02
	32,736	3.16
	49,104	5.20
8.184	8,184	0.32
	16,368	1.17
	24,551	1.37
4.092	4,092	0.16
	8,184	0.31
	12,275	0.46
